# Endovascular treatment of carotico-cavernous fistulas with acrylic glue: a series of nine cases

**DOI:** 10.1007/s00234-016-1760-4

**Published:** 2016-10-29

**Authors:** Marcus Ohlsson, Arturo Consoli, Georges Rodesch

**Affiliations:** 1 Service de Neuroradiologie Diagnostique et Thérapeutique, Hôpital Foch, Suresnes, 92151 France; 2 Department of Neuroradiology, Karolinska University Hospital, 171 76 Stockholm, Sweden

**Keywords:** Carotico-cavernous fistula, NBCA, Ehlers-Danlos, Collagen disease, Cavernous sinus

## Abstract

**Introduction:**

Injuries to the internal carotid artery close to the cavernous sinus may result in a fistulous connection between the artery and the venous sinus. Symptoms include pulsatile tinnitus, intracranial bruit, ophthalmological symptoms, and risk of intracerebral hematoma in cases of cortical venous reflux. Previous treatment strategies have included detachable latex balloons, coils, covered stents, or combinations thereof. Today, detachable latex balloons are phased out or withdrawn from several markets. Acrylic glue is a proven stable material used for embolization of arteriovenous shunts. It is a precise, fast, and cost-effective method of endovascular embolization, and it does not cause artifacts on MRI or MRA.

**Methods:**

We treated nine patients suffering from direct fistulas with acrylic glue without any permanent neurological adverse events.

**Results:**

Four patients were treated with glue embolization of the fistula without occlusion of the parent artery. Five patients with long-lasting symptomatology, large tears in the ICA, and with full collateral cerebral circulation were treated with glue embolization of the fistula and sacrifice of the ICA antero- and retrograde via the ICA and the posterior communicating artery.

**Conclusion:**

We suggest acrylic glue to be added to the panel of embolic materials used to treat CCFs.

## Introduction

Injuries to the internal carotid artery at or close to the cavernous sinus may result in a fistulous connection between the artery and the venous sinus. Symptoms created by such high-flow carotid-cavernous fistulas (CCFs) may differ, depending on the pattern of the venous drainage. Pulsatile tinnitus with objective intracranial bruit and ophthalmological symptoms as exophthalmos, conjunctival hyperemia, glaucoma, oculomotor nerve dysfunctions with diplopia, are the most frequent symptoms reported. Intracranial hemorrhage may occur, due to reflux into cortical venous contributors of the cavernous sinus and overload and/or rupture of these venous channels.

Depending on the underlying arteriovenous connection, carotico-cavernous fistulas have been classified in four different groups according to Barrow et al. [1]. Type A fistulas are direct ICA-CC fistulas following trauma or aneurysm rupture, whereas type B is between meningeal branches of the ICA and the cavernous sinus; type C arise from ECA branches, most often middle meningeal or accessory meningeal arteries; and type D have arterial supply from both ICA end ECA branches. We will consider in this report only direct type A fistulas arising from either trauma or spontaneous due to collagen disease with rupture into the cavernous sinus.

As a pathological entity known since the early 1900s [2], various treatments have been suggested for CCFs. Until the advent of endovascular routes in the 1970–1980s, surgical sacrifice of the ipsilateral ICA was most often considered standard. The results from such ICA sacrifice are not encouraging in modern practice; successful treatment was only achieved in 52 % of cases with mortality of almost 6 % and hemiplegia in another 13 % [2]. Direct closure of the fistula via open surgery was considered risky and not advisable, albeit tried with varying results.

With the development of endovascular techniques, alternative treatments have been performed via both intraarterial and intravenous approaches [3–7], using a variety of embolic materials, as detachable latex balloons, coils, covered stents, or combinations of these methods. Detachable latex balloons have been considered as the most appropriate tool to occlude a CCF because of their safety of use and affordable price. They are unfortunately now in limited supply and phased out or withdrawn from several markets. Coils, covered stents, and flow diverters are expensive methods and not always available in low- or medium-income countries. Furthermore, the use of a material requiring subsequent antiplatelet treatment may represent a challenge in a traumatized patient in need of emergency treatment. There is therefore a place for other emboli: acrylic glue is a proven stable material used for embolization of arteriovenous shunts since a long time and is furthermore a cheap, fast, and cost-effective method of endovascular embolization.

Patients with collagen disease as Ehlers-Danlos syndrome or fibromuscular dysplasia have dissection-prone vessels and are more at risk to iatrogenic vascular injury during endovascular surgeries [8]. Extensive endovascular procedures requiring multiple passes and repositions of wires and catheters may carry a prominent risk and should therefore be limited. We share here our experience and detail the techniques used for safe and proper treatment, and report our indications for glue embolization. We report in this paper nine cases of endovascular treatment of type A CCFs using acrylic glue (Glubran® or Histoacryl®) only, resulting in successful obliteration of the fistula or, when so desired, safe and permanent closure of the ICA.

## Materials and methods

We retrospectively reviewed the files of nine patients suffering of direct CCFs referred to one of us (GR) and managed endovascularily. These patients, average age of 35 years old at time of surgery, range 16–86 years, 5 male and 4 female, were included in the study (Table 1). Four patients had signs of collagen dysplasia, out of which one had confirmed Ehlers-Danlos disease. Five patients had suffered previous head trauma. Six patients were diagnosed, treated, and followed at the same hospital. Three patients were foreign referrals to our center, and as such, diagnosed and followed after treatment in their respective country of origin.

**Table 1:** Patients included

Age	Gender	Cause	Main complaint	Duration of symptoms	Technique
24	Male	Unknown	Bruit, ocular nerve palsy	1 year	ICA sacrifice
50	Female	Unknown	Venous congestion, exophthalmus	3 years	ICA sacrifice
25	Male	Trauma	Head trauma	8 months	ICA sacrifice
21	Male	Trauma	Exophthalmus, bruit	6 years	CCF closure
86	Female	Unknown	Exophthalmus, bruit	4 months	CCF closure
33	Female	Ehlers-Danlos	Bruit, eye hyperemia	4 months	CCF closure
16	Male	Trauma	Head trauma	1 month	CCF closure
18	Male	Trauma	Head trauma	5 months	ICA sacrifice
44	Female	Trauma	Head trauma	10 months	ICA sacrifice

Nine patients, five male and four female aged 16–86 years were included. Age (years), underlying cause (if known) of fistula, main complaint at presentation, duration of symptoms and type of treatment.

Initial radiological examination included MRI and MRA, followed by catheter angiogram under general anesthesia at the beginning of the treatment session. Four patients were treated with glue embolization of the fistula without sacrifice of the parent artery. Five patients with long-lasting symptoms, large tears, and with an angiogram demonstrating adequate cerebral collateral circulation were targeted for treatment with sacrifice of the internal carotid artery.

### Embolization technique

Using standard transfemoral Seldinger technique, diagnostic angiographic workup was performed including selective injections of the ICA, ECA, and VA bilaterally in standard antero-posterior (AP) and lateral views as well as 3D rotational angiography. This allowed to depict properly the localization of the fistulous hole, the potential collateral supply to the brain and the venous drainage pattern of the lesion.

After assessment of the anatomy and architecture of the CCF, according to the patient’s clinical status and the length of duration of the symptoms, it was decided to either selectively occlude the fistula (if the tear in the carotid artery was considered to be small) or to sacrifice the carotid artery endovascularily (if the fistulous communication was large) in patients with a long-lasting medical history of CCF and related symptoms.

### Selective occlusion of the CCF

**Fig. 1:**
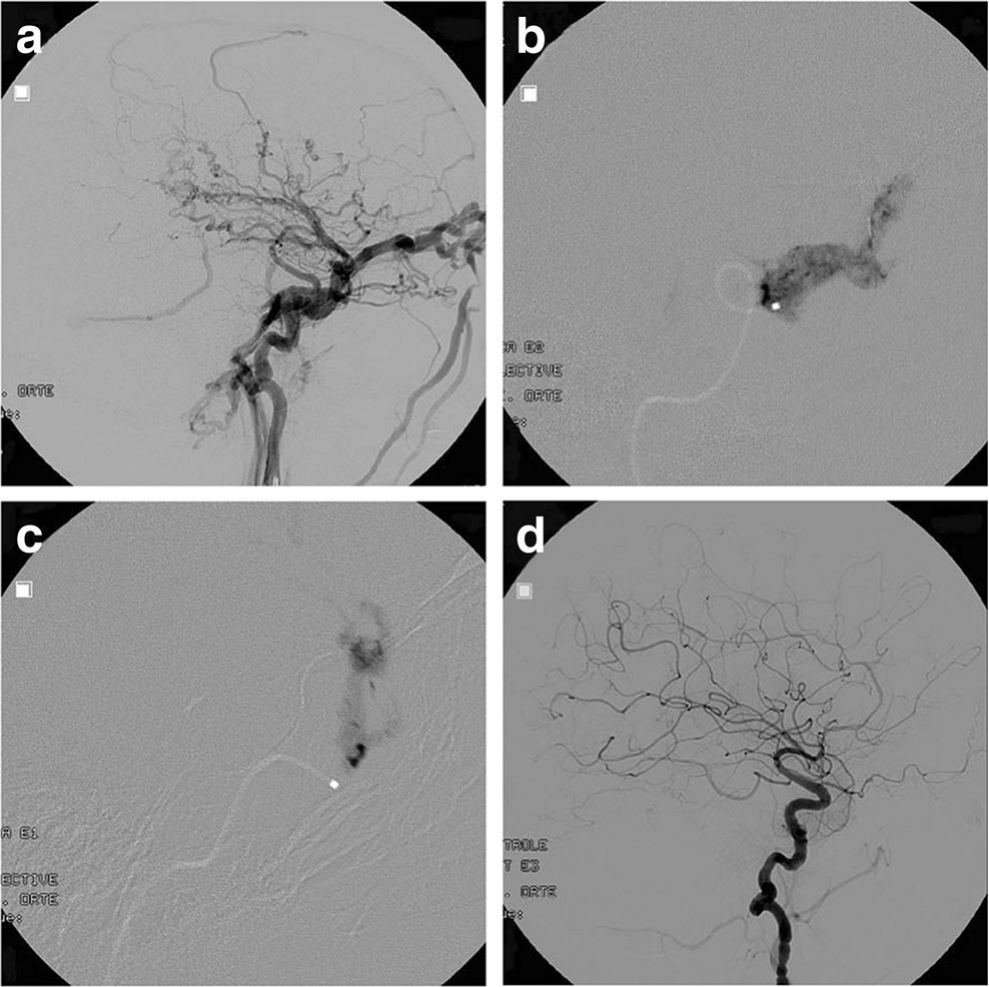
33-year old female patient with Ehlers-Danlos syndrome and right-sided spontaneous direct CCF, presenting with pulsatile exophthalmus, conjunctival hyperemia and history of a right-sided temporal hematoma. **a** Right ICA injection demonstrating a direct CCF with cortical venous reflux as superior ophthalmic artery and inferior petrosal sinus drainage. **b**, **c**: Three precise NBCA injections (two are here shown) into the cavernous sinus at the point of drainage of the fistula seals it shut. **d** Post-operative right ICA injection. The fistula is obliterated and the ICA kept patent. No distal emboli are seen and the cortical venous reflux is suppressed. The patient improved and normalized after embolization.

**Fig. 2:**
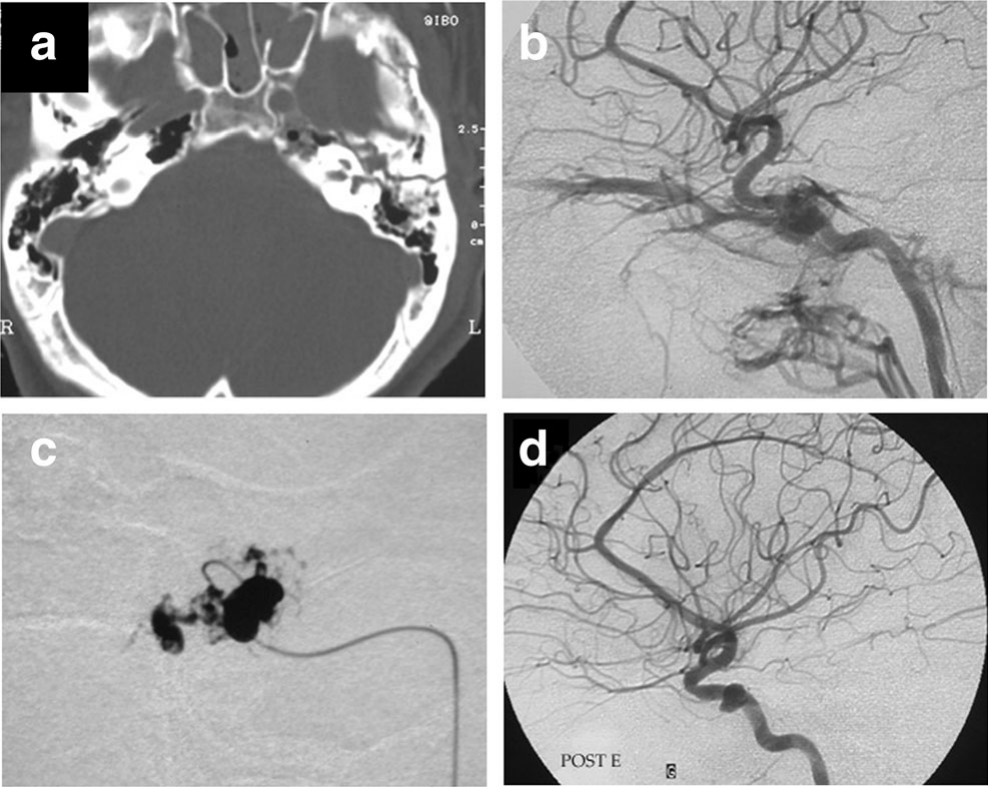
16-year old male patient suffering a complex compound skull base fracture after a motor vehicle accident (CT-scan, **a**), presenting with 1-month history of pulsatile exophthalmus. **b** Initial DSA demonstrates high-flow CCF with prominent venous hypertension. **c** With a precise NBCA injection in the cavernous sinus at the fistulous point, the CCF is closed. **d** Late follow-up control angiogram demonstrating full occlusion of the CCF, patent ICA, and the classical pouch often seen at the fistulous communication after cure. The small residual pouch seen in panel **d** did not warrant further treatment.

**Fig. 3:**
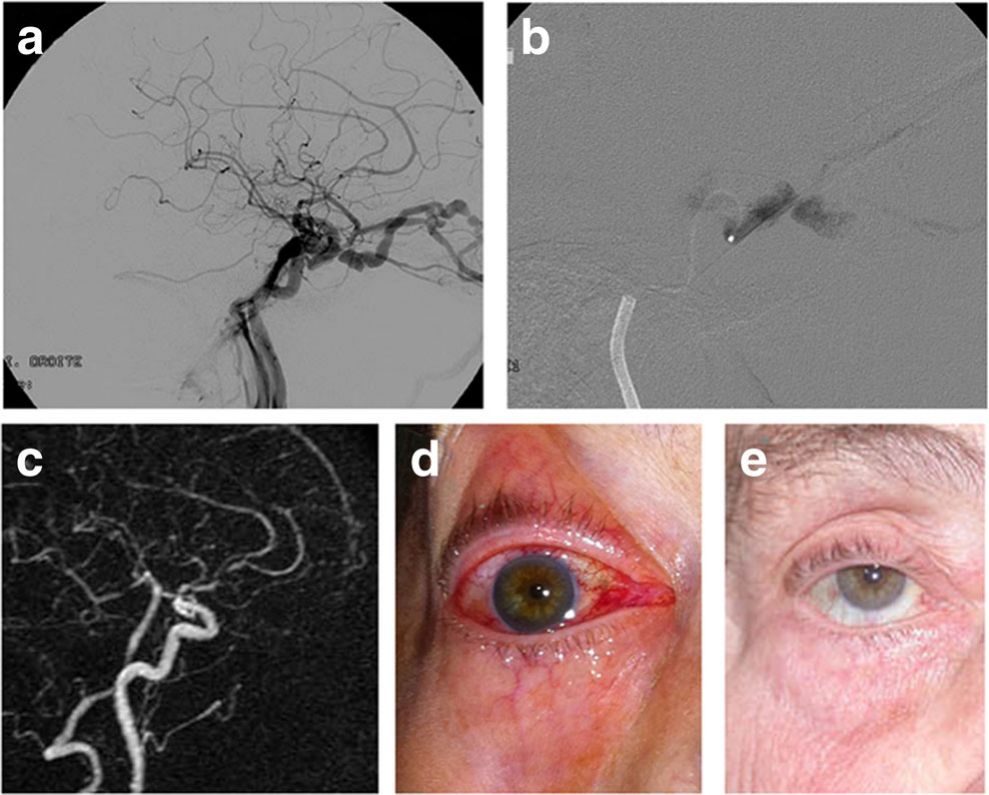
86-year old lady with a 4-month history of conjunctival hyperemia and intracranial bruit without any known triggering factor. **a** Angiogram reveals a high-flow right-sided CCF draining towards the inferior petrosal sinus and ophthalmic veins responsible for orbital venous congestion (**d**). **b** A targeted NBCA injection at the fistulous point in the cavernous sinus obliterated the fistula. **c** 3-month post-operative MRA demonstrating patency of the ICA and disappearance of the pathological venous drainage. Clinical appearance of the right eye prior to (**d**) and at 24-h post-embolization (**e**).

A 6F Envoy guiding catheter (Johnson-Johnson Codman, Raynham MA) was placed in the ICA and a microcatheter (Magic 1.8 or Baltacci 1.8, Balt, Montmorency, France) was inserted with the help of a microguide wire (Hybrid 008, Balt, Montmorency, France, or Mirage 008, Microtherapeutics, Irvine, CA) in the cavernous sinus through the fistulous communication. Selective injections were performed in order to assess the venous compartment in which the catheter was placed. Furthermore, this allowed to test the stability of the catheter’s position. Indeed, it was considered important to obtain a secure position of the tip of the microcatheter, close or against to the wall of the cavernous sinus in order to obtain a safe deposition of glue without reflux in the parent artery. This position of the microcatheter tip allows the first drop of glue to stick to the wall of the cavernous sinus, avoiding any important spilling of glue or erratic embolus. When this could be achieved, the microcatheter was purged with 5 % glucose, the acrylic glue (2 cm^3^: Histoacryl, Braun Melsungen, Germany, or Glubran (GEM, Viareggio, Italy) was added to a Lipiodol (0.2 cm^3^: Guerbet, France) and the tantalum powder (0.5 g: Balt, Montmorency, France) mixture was slowly injected under angiographic runs at 3 frames/s within the cavernous sinus compartment draining the fistula. The slight lag in between acquisition and presentation of the runs was here insignificant. Live road map was not used as it does not produce the same picture sharpness as a 3-fps run does. The Glubran®: Lipiodol® mixture rapidly polymerized in the cavernous sinus occluding the fistulous point from the venous side, resulting in occlusion of the CCF. The micro- and intermediate catheters were retrieved when retrograde glue reached the tip of the microcatheter in order to avoid it to be stuck, which never occurred in our experience. Control angiograms were obtained in order to assess either the cure of the lesion or the need for further embolization via the same technique. For illustration, please see Figs. 1, 2, and 3.

### Sacrifice of the ICA with CCF

**Fig. 4:**
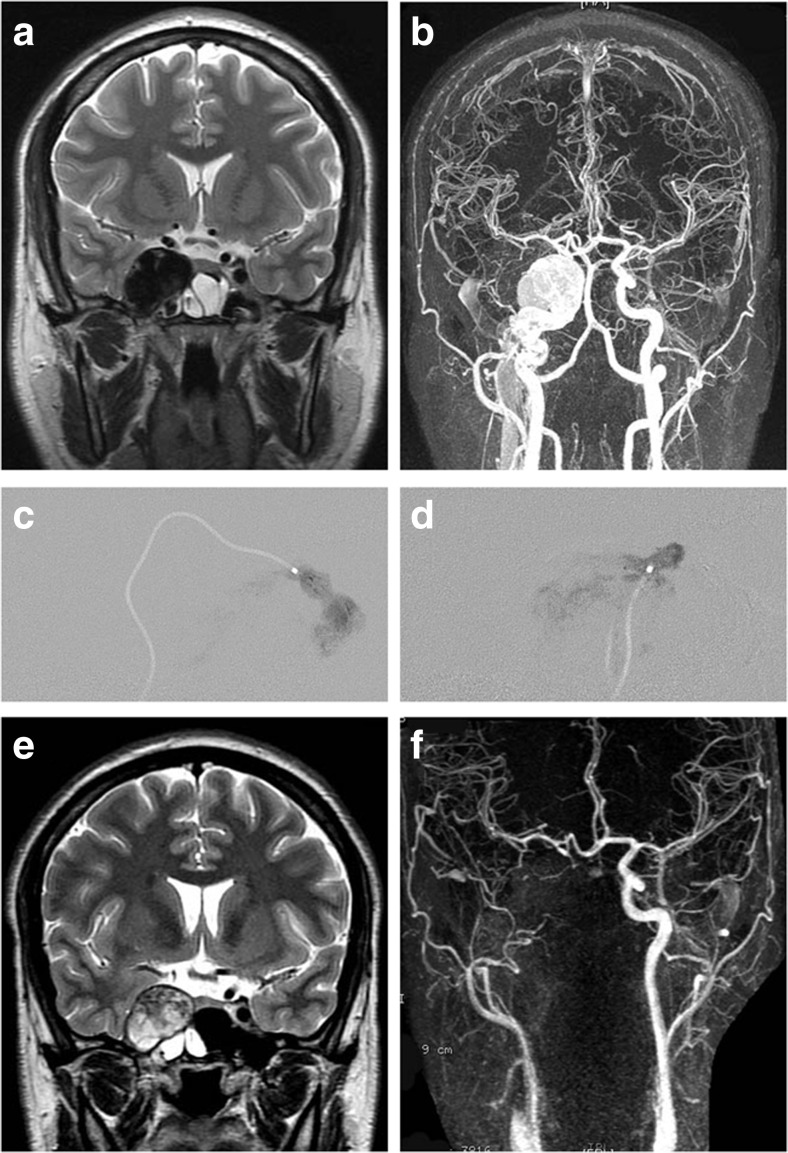
24-year old male patient with no clear head trauma recorded presented with a year-long history of pulsatile tinnitus and right sixth nerve palsy. **a**, **b** Preoperative MRI and MRA depicting right-sided CCF and a dilated cavernous sinus. DSA (not shown) excluded a ruptured intracavernous carotid aneurysm. It confirmed a CCF with a large tear in the ICA, draining posteriorly to the inferior petrosal sinus, with no direct supply to the ipsilateral hemisphere but adequate collateral circulation via anterior and posterior communicating arteries. Because of the suspicion of collagen disease and subsequent risks of balloon manipulation, it was decided to occlude the CCF by sacrificing the ICA. Glue injections were performed via the posterior communicating artery (**c**) and the intracavernous segment of the ICA (**d**), which sealed the CCF and occluded the ICA. **e**, **f** 1-month post-operative MRI and MRA demonstrating obliteration of the fistula and ICA. The patient reported full remission of symptoms. Note absence of MRI/MRA imaging artifacts after treatment with NBCA.

**Fig. 5:**
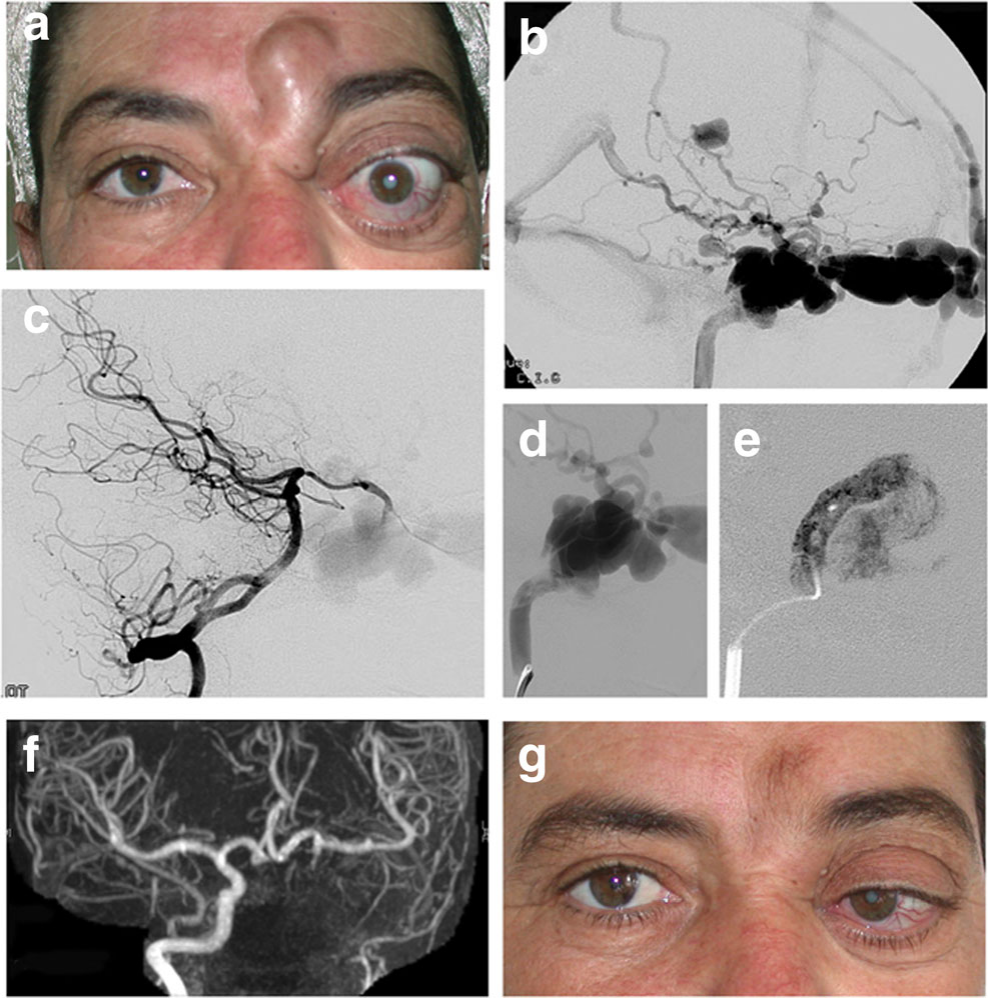
50-year old female patient without previous history of head trauma presented with 3-year history of secondary orbital and facial venous congestion (**a**) related to a left-sided high-flow CCF draining towards the superior ophthalmic vein, as superficial and deep cortical veins (**b**). Left vertebral artery injection (**c**) and left internal carotid artery injection (**d**) confirmed the fistulous communication. Because of the suspicion of underlying collagen disease and the risks of balloon manipulation, the circulation to the ipsilateral hemisphere being brought by collateral circulation, it was decided to sacrifice the carotid artery and occlude the fistula by glue injections via the internal carotid (**e**) and the posterior communicating arteries (not shown). **f**: post-operative MRA 24 h after embolization confirmed obliteration of the fistula and ICA with collateral flow to the left hemisphere via the anterior communicating artery. **g** Orbital and facial venous dilatations resolved after treatment (clinical appearance 5-day post-embolization), a slight 6th nerve palsy was present, which fully resolved after a few additional days.

This has been performed in high-flow cases with large lacerations of the carotid artery, giving rise to absence of antegrade flow of the ICA towards the brain, vascularization of the ipsilateral hemisphere via the contralateral ICA and the vertebral artery through anterior and posterior communicating arteries. In these cases, the CCF was also filled by a retrograde flow into the supraclinoid carotid segment. It has been then decided to occlude the shunt and the segment of the traumatized carotid artery. Using the same material for endovascular approach, the tip of the microcatheter was left in the carotid artery close to the fistulous point: the same concentration of glue was then injected under angiographic runs at 3 frames/s in order to occlude both the shunt (allowing glue to penetrate through the fistulous hole inside of the cavernous sinus) and the carotid by reflux towards the catheter tip. The catheters were removed when retrograde glue reached the distal end of the microcatheter. If this embolization proved not to be curative on the control angiogram, a second glue injection was performed after catheterization of the posterior communicating artery in the same conditions and achieved to trap the fistula and occlude the ICA. For illustrations, please see Figs. 4 and 5.

All embolized patients were left for some days under corticosteroids in order to reduce the inflammatory effect related to the polymerization of the glue. No heparin was used during or after the procedure, as we did not see significant flow reduction in any of the adjacent veins draining to the cavernous sinus. No intracranial adverse advents, no permanent oculomotor nerve palsy or ophthalmoplegia were noted at or immediately after embolization. One patient (Fig. 5) presented transient sixth nerve palsy, with only slight and short-lasting subjective deficits, which spontaneously resolved a few days after treatment. One patient with Ehlers-Danlos syndrome suffering of spontaneous CCF had a groin hematoma at the site of access, which did not warrant any active treatment but compression at the puncture point.

## Results

In this study, we treated nine patients suffering from Barrow type A direct fistulas with acrylic glue. All nine patients had immediate closure of the fistula at the end of the treatment session, as intended. Four patients were treated with glue embolization of the fistula without occlusion of the parent artery. Five patients with long-lasting symptomatology, large tears in the ICA, and with an angiogram demonstrating full collateral circulation were treated with glue embolization of the fistula and sacrifice of the ICA anterograde via the ICA and retrograde via the posterior communicating artery. One patient with confirmed Ehlers-Danlos syndrome had a minor hematoma at the groin site of access, however not warranting any further clinical procedure other than follow-up. No signs of distal embolization, cerebral ischemia, or hemorrhage were seen in any procedure. Besides from the single case of groin hematoma and the one case with transient sixth nerve palsy, no adverse events were seen. No permanent oculomotor deficits were noted.

Six patients had an angiogram and/or MRI/MRA at 2 to 6 months after treatment, all without any signs of recurrence or recanalization of the fistulous lesion. Imaging artifacts on MRI from the glue/tantalum injections were absent (Figs. 3, 4 and 5). All patients reported significant improvement of clinical symptoms with regression of eye symptoms and/or decreased or absent cranial bruit. Three referred foreign patients had clinical follow-up in their respective country of origin and to date, no reports of recurrence or adverse events have been communicated to the authors.

## Discussion

Treatment of CCFs has evolved considerably from the first treatment reported in 1809 when ligation of the CCA was done [2]. Sacrifice of the ICA was one of the first reported strategies for CCF, and the method may still be considered as a treatment modality in selected rare cases [2, 9].

The first reports of modern endovascular treatment for CCFs were in the early 1970s by using detachable latex balloons [10]. Endovascular treatment modalities evolved with the advent of detachable coils in the 1990s and was developed further with stents, Onyx, and off-label use of vascular plugs until present day. Although used for other arteriovenous fistulas, radiosurgery was never a good option for high-flow CCFs but has in some centers held an adjunct treatment modality for low-flow Barrow B-D type fistulas [2, 9, 11].

Detachable latex balloons, previously the mainstay in treatment of CCF, are easy to maneuver, affordable, and supple to use with advantages in wall remodeling close to the fistula. However, despite these positive advantages, latex balloons are now being progressively phased out and withdrawn and no longer accessible in all markets. Albeit still accessible in limited supply, the balloons may deflate over time and the method sometimes calls for additional coiling and/or injection of embolic material like Onyx or NBCA [4, 12]. Latex balloons cannot be combined with the here-described glue embolization technique, as glue will cause the balloon to rupture. Latex is also a material which in some individuals may cause allergic reactions, limiting its use in such cases.

Direct coiling, balloon- or stent-assisted coiling are nowadays techniques readily available in most neurointerventional centers. The technique is previously described [3] and will in most cases be sufficient, and is advocated in cases of aneurysmal rupture as underlying cause for the CC-fistula. However, coil compaction with subsequent risk of recanalization still constitutes a caveat to this technique. Coiling of the cavernous sinus may be done both from the arterial and venous route. The latter has often been used in indirect dural fistulas via the inferior petrosal sinus, superior petrosal sinus, facial veins, or the superior ophthalmic vein [13, 14], having demonstrated obliteration rates up to 90 % [14]. Direct fistulas from the ICA to the cavernous sinus can also be approached and treated transvenously. The technique may be considered if the arterial route is inaccessible or considered complicated, for example in Ehlers-Danlos patients [15].

Flow diverters and covered stents have gained interest as devices suitable to treat CCFs [16, 17]. However, besides being expensive, they require antiplatelet treatment for at least 3–6 months, sometimes life-long, with subsequent risk of adverse events before the device is endothelialized. These devices may by some centers be considered as first-line treatment, however not without caveats. Any antiplatelet regimen is risky in patients with recent multitraumas with or without traumatic brain injury. Also, there have been reports of failure of covered stents resulting in induced fistulas requiring additional treatment with Onyx [18]. Onyx is another liquid embolus and could be used for similar managements. We do not have any experience with this material in these diseases and therefore cannot comment further properly about its position in treatment of these fistulas. Abstaining from implantable devices and Onyx may in addition also result in less imaging artifacts later on, which is a factor to consider when follow-up is planned in a center not having access to Vaso-CT or Dyna-CT.

Stemming from experience in treatment of high-flow AVMs and other intracranial fistulas, glue has had a proven treatment record and is today still used to treat these lesions successfully [8]. A CCF is a skull base arteriovenous fistula and could therefore be approached using the same methodology. As for any other AV shunt, pre-therapeutic precise analysis of the regional and lesion anatomy is warranted. The cavernous sinus is anatomically not a sinus proper; it closely resembles a venous plexus where different compartments are separated by thin walls and trabecles [8, 19]. A CCF thus usually drains into one of the compartments and occlusion of that precise compartment will not create occlusion of the whole cavernous sinus. It may remain patent and continue to drain the brain properly after embolization. Therefore, the venous compartment draining the CCF and the brain have thus to be recognized and understood so that selective occlusion of the former can be properly achieved without impairing the latter. This also explains why permanent ophthalmological deficits rarely are seen after glue injection into the cavernous sinus. The inflammatory effect of the polymerizing glue as sudden changes in venous drainages (sludge and thrombosis) may however cause transient nerve palsies, as seen in the case in Fig. 5, which normally regress within a few weeks under steroid therapy.

The arterial side, the ICA, also needs special attention, and the understanding of the architecture of the fistulous communication is important for proper therapeutic management (whatever the technique used). Using glue to close CCF represents a crucial moment when the fistula closes, as the flow could be directed into the still patent ICA with risk of distal glue embolization with possible detrimental consequences [17]. It is the reason why we have chosen to inject the glue under angiographic runs at 3 frames/s so that precise visualization of the glue can be obtained.

Should there be a large-size fistula and given that adequate perfusion of the ipsilateral hemisphere may be provided via patent collateral circulation, endovascular sacrifice if the ICA can be considered. Previously, sacrifice was made via CCA or ICA ligation, which was followed by detachable latex balloons or off-label use of Amplatzer vascular plugs [12] if needed. However, detachable latex balloons have been reported to manage to preserve the patency of the ICA in about 70 % of cases [8]. The five cases of our series show that proper injections of glue in the ICA can result in sacrifice of the internal carotid artery and occlude the CCF, with minimal risk of adverse distal embolization and no noted recanalization. We believe this approach with supple microcatheters and glue can be considered as an alternative to techniques using comparably stiffer microcatheters for coils and stents. The proposed technique may also be done from the contralateral ICA via a patent anterior communicating artery or via the vertebrobasilar circulation and the posterior communicating artery, which may be of interest if the proximal ICA for any reason is inaccessible [20].

Although not within the scope of the present report, one may also combine glue injections with coiling by first placing coils and then occluding the fistula with glue. This approach may facilitate glue injection, thanks to the reduction of the flow obtained with coils [6].

In cases of known connective tissue disease with higher risk of iatrogenic injuries, for instance in Ehlers-Danlos syndrome with elevated risk of CCF [21, 22], special care must be taken to insure a safe endovascular procedure. Some centers may still advocate open surgery for these patients, and there have been reports of combinatorial approaches with surgical exposure of the cavernous sinus followed by direct insertion of endovascular sheaths and embolization [23]. In order to avoid accidental dissections, rapid and less traumatic techniques are here advisable as we seek to limit both the amount of hardware and the duration of endovascular intervention in cases of spontaneous CCFs. Balloons require inflation, deflation, and detachment with pulling on the catheters, all risks of further damage to the ICA. Coils may require multiple repositions of the catheter and several catheterizations of the fistula if kickback of the catheter occurs during coil deployment—all adding risks to the carotid wall. As the described technique only requires a minimum of catheter manipulations, preferably a precisely placed supple flow-directed microcatheter such as the here-employed Magic® and almost instantaneous occlusion of the fistula with precise injection of glue, we believe this approach as safe in regards to accidental preoperative dissection, however of course not without such risk. Here, we report one successfully treated CCF in an Ehlers-Danlos patient with glue embolization.

Today, endovascular devices are still expensive and the cost of hardware is an ever-present factor in any clinical practice and also limits the use of neurointerventional strategies in low-income settings. Glue embolization could be of value as less costly treatment method in both developing economies as in any modern hospital setting. We believe the method presented here, if used by experienced interventional neuroradiologists, is as safe and efficient as any device-driven modality. Previous experience from glue embolization of fistulas or AVMs is a prerequisite and not having necessary knowledge constitutes a limitation of the technique in the interest of safe medical practice.

The methods here presented are novel use of classical techniques, using proven methodology and materials. We demonstrate adequate closure of Barrow type A fistulas without any permanent neurological adverse events. In all, we suggest that, when properly used, glue can be added to the panel of embolic materials used to treat CCFs.

## Abbreviations

CCA: Common carotid artery
CCF: Carotico-cavernous fistula
DSA: Digital subtraction angiography
ECA: External carotid artery
ICA: Internal carotid artery
MRI: Magnetic resonance imaging
MRA: Magnetic resonance angiography
NBCA: n-Butyl cyanoacrylate
Onyx®: Ethylene vinyl copolymer and micronized tantalum powder in dimethyl-sulfoxide (Covidien)
VA: Vertebral artery
